# Risk of cervical intraepithelial neoplasia grade 3 or more diagnoses for human papillomavirus16/18-positive women by cytology and co-infection status

**DOI:** 10.1186/s13027-023-00540-9

**Published:** 2023-10-09

**Authors:** Mengyin Ao, Xiaoxi Yao, Danxi Zheng, Xuesai Gu, Mingrong Xi

**Affiliations:** 1 Department of Gynecology and Obstetrics, West China Second University Hospital, Sichuan University, Number 20, Third Section of People’s South Road, Chengdu, 610000 China; 2 Key Laboratory of Birth Defects and Related Diseases of Women and Children, Sichuan University, Chengdu, Sichuan China; 3 Department of Information Management, West China Second University Hospital, Sichuan University, Number 20, Third Section of People’s South Road, Chengdu, 610000 China

**Keywords:** HPV 16, HPV 18, Cervical cancer screening, CIN 3, Multiple HPV infection

## Abstract

**Background:**

Human papillomavirus (HPV) 16 and 18 cause approximately 70% of cervical cancer cases. The aim of this study was to evaluate whether co-infected with other HPV genotypes will affect the risk of cervical carcinogenesis in HPV16/18 positive-women.

**Methods:**

In this cross-sectional study, cervical cytology and histological classifications from women who tested positive for HPV 16/18 and underwent colposcopy within 6 months, between January 2010 and May 2021 were obtained from West China Second University Hospital of Sichuan University.

**Main outcomes and measures:**

Immediate risk of cervical intraepithelial neoplasia grade 3 or more diagnoses (CIN 3+).

**Results:**

A total of 7940 HPV 16/18-positive women were included, with a median age of 40 years (range 25–84 years). Among them, 2710 (34.1%) were infected with multiple genotypes, 6533 (82.28%) had cytology results and 2116 (26.65%) women were diagnosed with CIN 3+. The effects of HPV 16/18 coinfecting with other HPV on CIN3 + risk varied with specific HPV genotypes. After adjusting for cofactors, compared to single HPV 16 infection, the CIN 3 + risk was significantly reduced in women infected with HPV 16 + other high-risk HPV (hrHPV) [odds ratio (OR) = 0.621, 95% confidence interval (CI) 0.511–0.755], HPV 16 + low-risk HPV (lrHPV) (OR = 0.620, 95% CI 0.436–0.883), and HPV 16 + lrHPVs + other hrHPVs (OR = 0.248, 95% CI 0.157–0.391). The prevalence of CIN 3 + was associated with increased severity of cytologic abnormalities in HPV 16/18-positive women and peaked at cytology HSIL + (89.9% and 82.3%), which held a substantially greater risk than that of NILM (OR = 65.466, 95% CI 50.234–85.316).

**Conclusions:**

In this cross-sectional study of HPV 16/18-positive women, the effects of multiple infection were likely complicated and varied with specific HPV genotypes. The coinfection of HPV 16 and other genotypes of HPV except HPV 18 was associated with decreased CIN 3 + risk. Cytologic results were informative when HPV 16/18 was positive. It might be reasonable to recommend expedited treatment for patients with HPV 16/18 positive and HSIL + cytology in the Chinese population.

**Supplementary Information:**

The online version contains supplementary material available at 10.1186/s13027-023-00540-9.

## Background

Persistent human papillomavirus (HPV) infection is the main etiological factor for cervical cancer. HPV 16 and 18 cause approximately 70% of cervical cancer cases [1]. However, it is controversial whether co-infection status will influence the risk of cervical carcinogenesis in HPV 16/18-positive women. Since many studies have recommended HPV-based testing as primary screening method [2–5], research on this topic is essential.

In large studies of populations from China, the United States, Norway and Italy, the prevalence of multiple infection is 4.6–8.2%, accounting for 20.4–43.9% of HPV-positive women [6–10]. A better understanding of interactions among different HPV genotypes contributes a lot to the risk assessment of women with multiple HPV infection and might provide information for the development of the next generation of HPV vaccine via predicting the possible genotypes that interact with those targeted by current vaccines. However, there is no consensus on the effects of multiple HPV infection. Some studies showed that multiple infection increased the risk of cervical intraepithelial neoplasia grade 3 or more severe diagnoses (CIN 3 +) [9–11], and it was reported as the result of additive or synergistic effects [6, 8, 12]. Conversely, Ping Xu et al. [6] found that HPV 16 co-infected with other high risk HPV (hrHPV) was associated with a lower risk of CIN 3+. Besides, most studies did not distinguish HPV genotypes except for HPV 16 and 18, and thus the influence of specific HPV remains unclear.

The CIN 3 + risk of HPV 16/18-positive women with different cytologic results has been well assessed from the general screening population in the United States [13–15]. The American Society of Colposcopy and Cervical Pathology published a risk-based management consensus guideline in 2019, which suggested that the combination of HPV 16/18 genotyping and cytology permitted more precise management [2]. Nevertheless, limited data on the Chinese population is available [6, 16, 17]. Regardless of cytology results, colposcopy rather than expedited treatment remains the primary recommendations to HPV 16/18-positive women in China. It has been demonstrated that the prevalence of HPV genotypes in China is different from that in Western countries [18, 19]. Therefore, it is questionable whether the recommendations based on risk estimates of American women can be applied to the Chinese population.

In light of these facts, the study aimed to evaluate the effects of co-infection status and estimate the CIN 3 + risk for different cytology results in HPV 16/18-positive women.

## Methods

### Study design and participant enrollment

The Ethics Committee of West China Second University Hospital of Sichuan University (WCSUH) approved the protocol before the initiation of this investigation. Informed consent was waived because the study was an anonymous analysis of retrospective data. The study manuscript follows the Strengthening the Reporting of Observational Studies in Epidemiology (STROBE) reporting guidelines for cross-sectional studies.

A HPV genotyping examination with or without cytology was performed by gynecologists at WCSUH. The selection of cytology was determined by determined by the preference of clinicians and the specific condition of patients. Women who tested positive for HPV-16/18 were referred to colposcopy and biopsied if necessary. We recruited patients who tested positive for HPV 16/18 and underwent colposcopy within 6 months, between January 2010 and May 2021 at the outpatient department of WCSUH. The inclusion criteria were as follows: 25 years old or older; non-pregnant. Patients were excluded if they were immunocompromised or had a history of total hysterectomy or pelvic radiotherapy. We collected the following data from medical records: age, vaginal bleeding or not, results of HPV genotyping, cytology that obtained at the time of HPV genotyping, and pathological diagnoses. If patients had several HPV 16/18-positive results available, we collected the relevant information when the patients were positive for HPV 16/18 for the first time.

### HPV genotyping

HPV genotyping was performed by one of the following two methods: (1) the HPV 23 Genotyping Assay (YanengBIO, Shenzhen, China), which tests for 17 hrHPV types (HPV 16, 18, 31, 33, 35, 39, 45, 51, 52, 53, 56, 58, 59, 66, 68, 73, 82) and 6 low-risk HPV (lrHPV) types (HPV 6, 11, 42, 43, 81, 83) or (2) the Tellgenplex HPV27 Genotyping Assay (Tellgen, Shanghai, China), which tests for 17 hrHPV types (HPV 16, 18, 26, 31, 33, 35, 39, 45, 51, 52, 53, 56, 58, 59, 66, 68, 82) and 10 lrHPV types (HPV 6, 11, 40, 42, 43, 44, 55, 61, 81, 83) [19–23]. Both detection assays have been validated and approved by the CFDA. The method for each patient was chosen according to the preference of the patient and gynecologist. Testing procedures followed kit protocols provided by the manufacturers.

### Cytology

Cytology tests were performed using one of the following methods: the ThinPrep Pap test (histologic, Bedford, MA), or the SurePath Pap test (BD Diagnosis, Franklin Lakes, NJ). Samples were obtained by gynecologists and slides were prepared according to the manufacturers’ specifications. Cytology results were classified by WCSUH pathologists according to the 2001 Bethesda System terminology, including atypical squamous cells of undetermined significance (ASC-US), low-grade squamous intraepithelial lesion (LSIL), atypical squamous cells cannot exclude high-grade squamous intraepithelial lesion (ASC-H), atypical glandular cells (AGC, without subdivision), and high-grade intraepithelial lesion or worse (HSIL+). If two types of cytologic abnormalities (e.g. ASC-US and AGC-not otherwise specified, AGC-NOS) were detected in a sample, we would count both in during statistical analyses.

### Colposcopy-directed biopsy and histopathologic examination

All suspicious lesions under colposcopy were biopsied. Endocervical curettage (ECC) was performed when the cervical squamous column junction was unsatisfied. Histological results were obtained from colposcopy-directed biopsy, ECC, loop electrosurgical excision procedure (LEEP), cold knife conization (CKC), and hysterectomy. All pathological diagnoses were categorized into normal (including cervicitis), CIN 1, CIN 2, CIN 3, adenocarcinoma in situ (AIS), and cervical cancer. The diagnoses were based on the worst results if there were a range of severities. Some women with adequate colposcopy assessment and normal findings might not be biopsied and classified as normal.

### Statistical analyses

Histological results were classified as ≤ CIN 2 (including normal, CIN 1, and CIN 2) and CIN 3 + (including CIN 3, AIS, and cervical cancer). Variables were analyzed with $\chi^{2}$ test, Fisher’s exact test, and Kruskal–Wallis test, as appropriate. Logistics regressions were conducted to explore the CIN3 + risk of different HPV infection patterns and cytology results. A two-tailed *P* value < 0.05 was considered statistically significant. Statistical analyses were conducted using IBM SPSS statistics software (version 21.0; IBM Corp, Armonk, NY).

## Results

A total of 7940 HPV 16/18-positive women were included with a median age was 40 years (range, 25–84 years); 6211 (78.22%) and 1895 (23.87%) were positive for HPV 16 and 18, respectively. Among them, 34.1% (2710/7940) were infected with multiple genotypes, 82.28% (6533/7940) had cytology results and 26.65% (2116/7940) women were diagnosed with CIN 3 + (Additional file 1: Table S1). The prevalence of CIN 3 + in HPV 16-positive women (1899/6211, 30.57%) was significantly higher than that of HPV 18 (260/1895, 13.72%) (*P* < 0.001). The prevalence of CIN 3 + increased with age and differed significantly (Additional file 1: Fig. S1) (*P* < 0.001). The proportion of CIN 3 + in women with vaginal bleeding (316/588, 53.74%) was significantly higher than that in women without (1800/7352, 24.48%) (*P* < 0.001).

**Fig. 1:**
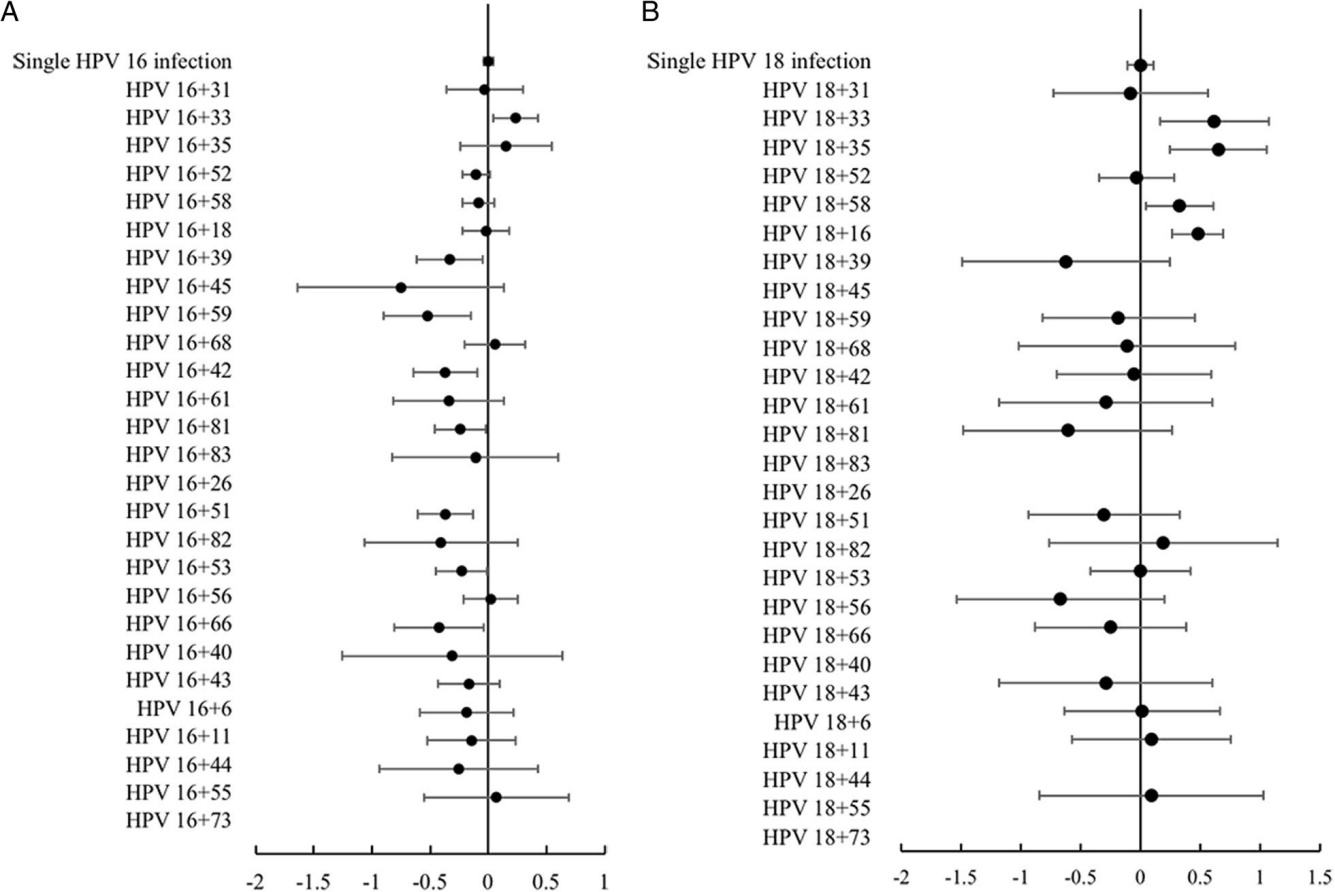
Log odds ratio for CIN 3 + in coinfection of HPV 16 (**A**) or HPV 18 (**B**) and other HPV genotypes. The analyses took single HPV 16 or HPV 18 infection as a reference, respectively. The vertical solid line represents the null log odds ratio of 0.HPV, human papillomavirus; CIN 3+, cervical intraepithelial neoplasia grade 3 or more severe diagnoses

Approximately 33.02% (2051/6211) of HPV 16-positive women had multiple infections, and that of HPV 18-positive women was 43.64% (827/1895). HPV 52, 58, 53, 81, 51, and 18 were common genotypes coinfecting with HPV 16, accounting for 22.09%, 16.43%, 11.21%, 10.24%, 9.36%, and 8.09%, respectively. HPV 18 often coinfected with HPV 16, 52, 58,53, 58, and 56, accounting for 17.91%, 16,72%, 11.43%, 9.17%, 8.95%, and 8.09%, respectively. Compared to single HPV 16 infection (Fig. 1A), HPV 16 + 33 was significantly associated with an increased risk of CIN 3 + (odds ratio [OR] = 1.722, 95% confidence interval [CI] 1.102–2.691). In contrast, HPV 16 + 39 (OR = 0.467, 95% CI 0.241–0.905), HPV 16 + 59 (OR = 0.301, 95% CI 0.127–0.712), HPV 16 + 42 (OR = 0.426, 95% CI 0.228–0.799), HPV 16 + 81 (OR = 0.575, 95% CI 0.348–0.950), HPV 16 + 51 (OR = 0.428, 95% CI 0.248–0.739), HPV 16 + 53 (OR = 0.592, 95% CI 0.358–0.981), and HPV 16 + 66 (OR = 0.378, 95% CI 0.157–0.909) were significantly involved with a lower risk of CIN 3+. Compared to single HPV 18 infection (Fig. 1B), HPV 18 + 33 (OR = 4.112, 95% CI 1.443–11.720), HPV 18 + 35 (OR = 4.486, 95% CI 1.775–11.335), HPV 18 + 58 (OR = 2.110, 95% CI 1.098–4.054), and HPV 18 + 16 (OR = 3.028, 95% CI 1.851–4.952) were related to a higher risk of CIN 3+.

According to the presence of other hrHPV and lrHPV, the included women were classified into 12 groups (Table 1). The diagnoses were significantly different in HPV 16-positive groups (*P* < 0.001), while there was no significant difference in HPV 18-positive groups (*P* = 0.266) and HPV 16 + 18-positive groups (*P* = 0.068). Notably, the prevalence of CIN 3 + in single HPV 16 (1408/4160, 33.85%), single HPV 18 (149/1068, 13.95%), and HPV 16 + HPV 18 (27/82, 32.93%) were the highest among HPV 16, HPV 18 and HPV 16 + 18 positive groups, respectively.

**Table 1:** Distribution of HPV 16/18 infection patterns among the study population (n = 7940)

HPV16/18 infection patterns	No. (%)						*P* Value
	Normal	CIN 1	CIN 2	CIN3/AIS	Cancer	Total	
HPV 16 only	1959 (47.1)	373 (9.0)	420 (10.1)	937 (22.5)	471 (11.3)	4160 (100)	< 0.001
HPV 16 + other hrHPVs	584 (45.9)	176 (13.8)	171 (13.5)	272 (21.4)	68 (5.4)	1271 (100)	
HPV 16 + lrHPVs	177 (53.3)	41 (12.3)	43 (13.0)	49 (14.8)	22 (6.6)	332 (100)	
HPV 16 + lrHPVs + other hrHPVs	147 (52.1)	57 (20.2)	41 (14.5)	29 (10.3)	8 (2.8)	282 (100)	
HPV 18 only	688 (64.4)	152 (14.2)	79 (7.4)	65 (6.1)	84 (7.9)	1068 (100)	0.266
HPV 18 + other hrHPVs	253 (59.0)	80 (18.6)	44 (10.3)	39 (9.1)	13 (3.0)	429 (100)	
HPV 18 + lrHPVs	76 (67.9)	21 (18.8)	6 (5.4)	7 (6.3)	2 (1.8)	112 (100)	
HPV 18 + lrHPVs + other hrHPVs	71 (59.2)	30 (25.0)	12 (10.0)	6 (5.0)	1 (0.8)	120 (100)	
HPV 16 + HPV 18	34 (41.5)	7 (8.5)	14 (17.1)	13 (15.9)	14 (17.1)	82 (100)	0.068
HPV 16 + HPV 18 + other hrHPVs	22 (43.1)	9 (17.6)	6 (11.8)	11 (21.6)	3 (5.9)	51 (100)	
HPV 16 + HPV 18 + lrHPVs	9 (60.0)	5 (33.3)	1 (6.7)	0 (0.0)	0 (0.0)	15 (100)	
HPV 16 + HPV 18 + lrHPVs + other hrHPVs	8 (44.4)	5 (27.8)	3 (16.7)	2 (11.1)	0 (0.0)	18	

*HPV* human papillomavirus, *CIN* cervical intraepithelial neoplasia, *AIS* adenocarcinoma in situ, *other hrHPVs* high-risk HPV 26/31/33/35/39/45/51/52/53/56/58/59/66/68/73/82, *lrHPVs* low-risk HPV 6/11/40/42/43/44/55/61/81/83

The cytology results of 6533 included women were available (Table 2). Most (2932, 44.88%) had NILM cytology, 1514 (23.17%) had ASC-US, 906 (13.87%) had LSIL, 334 (5.11%) had ASC-H, 151 (2.31%) had AGC and 770 (11.79%) had HSIL+. As expected, the prevalence of CIN 3 + generally increased with the severity of cytologic abnormalities in HPV 16-positive women: NILM (11.4%), ASC-US (23.2%), LSIL (28.8%), ASC-H (72.3%), AGC (66.0%) and HSIL + (89.9%). Results in HPV 18-positive women showed a similar trend, and it was noteworthy that HPV 18-positive women with AGC had a relatively high prevalence of CIN 3 + (72.0%).

**Table 2:** CIN 3 + risk of different cytology results in HPV 16/18-positive women (n = 6533)

HPV genotype	Cytology	CIN 3 + cases, No. (%)	Total	*P* Value
HPV 16	NILM	248 (11.4)	2185	< 0.001
	ASC-US	268 (23.2)	1157	
	LSIL	205 (28.8)	713	
	ASC-H	211 (72.3)	292	
	AGC	70 (66.0)	106	
	HSIL +	623 (89.9)	693	
HPV 18	NILM	38 (4.8)	799	< 0.001
	ASC-US	40 (10.3)	388	
	LSIL	23 (10.7)	214	
	ASC-H	17 (34.7)	49	
	AGC	36 (72.0)	50	
	HSIL +	79 (82.3)	96	

*HPV* human papillomavirus, *CIN 3* + cervical intraepithelial neoplasia grade 3 or more severe diagnoses, *NILM* negative for intraepithelial lesions or malignancy, *ASC-US* atypical squamous cells of undetermined significance, *LSIL* low-grade intraepithelial lesion, *ASC-H* atypical squamous cells cannot exclude high-grade squamous intraepithelial lesion, *AGC* atypical glandular cells, *HSIL* + high-grade squamous intraepithelial lesion or worse

To further investigate the association between the abovementioned factors and CIN 3 + risk, we conducted two logistic regression analyses which took single HPV 16 infection (Table 3) and single HPV 18 infection as reference (Additional file 1: Table S2), respectively. Older age women (OR = 1.017, 95% CI 1.011–1.024) and those with vaginal bleeding (OR = 2.674, 95% CI 2.113–3.384) were at higher risk of CIN 3 + independently. HPV 16/18-positive women with cytologic abnormalities also had a higher risk of CIN 3+. Of note, HSIL + (OR = 65.466, 95% CI 50.234–85.316), ASC-H (OR = 17.339, 95% CI 13.223–22.736), and AGC (OR = 14.963, 95% CI 9.562–24.413) were associated with a substantially greater risk of CIN 3 + than that of NILM. As demonstrated in the logistics regression analysis using single HPV 16 infection as reference (Table 3), HPV 16 + other hrHPVs (OR = 0.621, 95% CI 0.511–0.755), HPV 16 + lrHPVs (OR = 0.620, 95% CI 0.436–0.883), and HPV 16 + lrHPVs + other hrHPVs (OR = 0.248, 95% CI 0.157–0.391) were associated with decreased risk of CIN 3 + . Meanwhile, regardless of the HPV infection patterns of HPV 18 infected women, the CIN 3 + risk of HPV 18 infection was lower than that of HPV 16 infection: single HPV 18 (OR = 0.327, 95% CI 0.255–0.420), HPV 18 + other hrHPVs (OR = 0.229, 95% CI 0.153–0.342), HPV 18 + lrHPVs (OR = 0.315, 95% CI 0.149–0.665) and HPV 18 + lrHPVs + other hrHPVs (OR = 0.210, 95% CI 0.094–0.467). However, there was no significant difference in CIN 3 + risk between single HPV 16 infection and HPV 16 + HPV 18 (OR = 0.747, 95% CI 0.364–1.534), HPV 16 + HPV 18 + other hrHPVs (OR = 0.710, 95% CI 0.322–1.562), or HPV 16 + HPV 18 + lrHPVs + other hrHPVs (OR = 0.370, 95% CI 0.076–1.801). The results of logistic regression analyses using single HPV 18 and HPV 16 infection as references were identical except for HPV infection patterns. Compared to single HPV 18 infection, the CIN 3 + risk did not differ significantly in HPV 18 + other hrHPVs (OR = 0.699, 95% CI 0.443–1.102), HPV 18 + lrHPVs (OR = 0.962, 95% CI 0.442–2.096) and HPV 18 + lrHPVs + other hrHPVs (OR = 0.641, 95% CI 0.280–1.468) (Additional file 1: Table S2).

**Table 3:** Binary logistic regression analysis of factors influencing the risk of CIN 3 + in HPV 16/18-positive women

Characteristics	CIN 3 + versus ≤ CIN 2
	OR (95% CI)
Age	1.017 (1.011–1.024)
Vaginal bleeding	
No	Reference
Yes	2.674 (2.113–3.384)
Cytology	
NILM	Reference
ASC-US	2.339 (1.948–2.808)
LSIL	3.477 (2.836–4.264)
ASC-H	17.339 (13.223–22.736)
AGC	14.963 (9.562–24.413)
HSIL +	65.466 (50.234–85.316)
HPV infection patterns	
Single HPV 16	Reference
HPV 16 + other hrHPVs	0.621 (0.511–0.755)
HPV 16 + lrHPVs	0.620 (0.436–0.883)
HPV 16 + lrHPVs + other hrHPVs	0.248 (0.157–0.391)
Single HPV 18	0.327 (0.255–0.420)
HPV 18 + other hrHPVs	0.229 (0.153–0.342)
HPV 18 + lrHPVs	0.315 (0.149–0.665)
HPV 18 + lrHPVs + other hrHPVs	0.210 (0.094–0.467)
HPV 18 + HPV 16	0.747 (0.364–1.534)
HPV 18 + HPV 16 + other hrHPVs	0.710 (0.322–1.562)
HPV 18 + HPV 16 + lrHPVs	NA
HPV 18 + HPV 16 + lrHPVs + other hrHPVs	0.370 (0.076–1.801)

*HPV* human papillomavirus, *other hrHPVs* high-risk HPV 26/31/33/35/39/45/51/52/53/56/58/59/66/68/73/82, *lrHPVs* low-risk HPV 6/11/40/42/43/44/55/61/81/83, *CIN 3* + cervical intraepithelial neoplasia grade 3 or more severe diagnoses, *CIN 2* cervical intraepithelial neoplasia grade 2, *NILM* negative for intraepithelial lesions or malignancy, *ASC-US* atypical squamous cells of undetermined significance, *LSIL* low-grade intraepithelial lesion, *ASC-H* atypical squamous cells cannot exclude high-grade squamous intraepithelial lesion, *AGC* atypical glandular cells, *HSIL* + high-grade squamous intraepithelial lesion or worse, *CI* confidence interval, *OR* odds ratio. *NA* not applicable

## Discussion

This study is one of the largest research on HPV 16/18-positive women. Research on the effects of co-infection status might be helpful to risk stratification of HPV 16/18 infected women and more detailed interventions can be accessible in the future. Data on the CIN 3 + risk of different cytology results in a large population may provide evidence for improving the screening strategies of China.

The proportion of multiple infection in HPV 16/18 infected cases was similar to a large population study in which 26.5% of HPV-positive women had multiple infection [24]. In HPV 16/18 infected women, multiple infection is a common phenomenon. Our data suggested that for HPV 16/18 positive women, regardless of co-infection status, the immediate CIN 3 + risk was greater than the US benchmark (greater than or equal to 4%) for referral to colposcopy [2]. Thus, it is reasonable for the referral to colposcopy in the Chinese HPV 16/18 positive population.

There is no consensus on the association between multiple HPV infection and CIN 3 + risk. Previously it was assumed that each HPV genotype contributes to the risk of cervical precancer or cancer independently [25, 26]. However, recent findings demonstrated that both antagonistic and synergistic interactions might exist among different HPV genotypes [6, 12, 27–30]. Chaturvedi et al. [12] reported that multiple HPV infection within the A9 species or oncogenic types increased the risk of cervical diseases in contrast to single HPV infection. Likewise, our results demonstrated that the co-infection of two genotypes belonging to A9 species, HPV 16 and HPV 33 was significantly associated with an increased risk of CIN 3+. Consistent with a large population-based study [6], women with HPV 16 + 18 and either none, other hrHPVs, or lrHPVs were at a higher risk of CIN 3 + than single HPV 18 infection, whereas single HPV 16 infection and HPV 16 + HPV 18 had a similar risk of CIN 3 + in our study. Therefore, there might be synergistic interactions between HPV 16/18 and specific HPV genotypes. Meanwhile, we cannot exclude the possibility that the risk of multiple HPV infection was similar to the sum of estimated risk from individual genotypes, because the study did not include single infections of other HPV genotypes except HPV 16/18. We also observed antagonistic interactions between HPV 16 and other HPV genotypes, including HPV 39, 59, 42, 81, 51, 53, and 66. Furthermore, through multivariate analysis adjusted for several cofactors, we found that single HPV 16 infection was associated with a greater risk of CIN 3 + than those who were simultaneously infected by lrHPVs, and/or hrHPVs except for HPV 18. These findings were in agreement with those of Wheeler et al. [31], Wu et al. [6], and Sundström et al. [32] The mechanism of antagonistic interactions among HPV genotypes remain unclear. It was proposed that intergenotypic competition might interfere with the progression to CIN3 + in women with multiple HPV infections, which perhaps involved the key stages of HPV infection process, such as binding receptors, utilization of host cell organelles, synthesis of viral DNA, and insertion of viral DNA into the host genome [27]. Another possible mechanism is relevant to the immune response, especially cross-protection [6, 27, 33]. Concurrent infection of multiple HPV genotypes may induce a more effective immune response than a single HPV infection [27], and perhaps the infection of a less carcinogenic or non-carcinogenic HPV genotype triggers the immune response in advance, thereby reducing the pathogenicity of subsequent infection of HPV 16/18 [6]. Overall, the effects of multiple HPV infection might depend on specific HPV combinations and further research is needed to elucidate the mechanisms.

Generally, our data demonstrated that cytologic results were informative when HPV 16/18 was positive. It is noteworthy that HPV 16/18-positive women with cytology HSIL + were at a very high risk of CIN 3 + (over 80%), showing an approximately 65 folds higher risk than cytology NILM. By contrast, Risk estimates from a subset of women in the Kaiser Permanente Northern California screening program identified HPV16 and 18 with HSIL + had immediate CIN 3 + risk of 60% and 30%, respectively [13]. The possible explanation for the variation is that most of the Chinese population, especially those in rural areas, do not have access to regular screening, leading to a relatively higher risk of CIN 3 + at the first visit. [34–36] Consequently, it might be reasonable to recommend treatment without biopsy for non-pregnant women 25 years or older with positive HPV 16/18 and cytology HSIL + in China. Unlike HSIL+, cytology ASC-H in HPV 16/18-positive women predicted different risks of CIN 3+: 72.3% for HPV 16 and 34.7% for HPV 18. Accordingly, HPV 16/18-positive with ASC-H might not require the same intensive management as HSIL+, especially HPV 18. Colposcopy might still be preferred for HPV 16/18-positive women with ASC-H cytology. Another important finding is that almost half of the included women had HPV 16/18 and cytology NILM, among which CIN 3 + was identified in 11.4% of HPV 16-positive women while 4.8% of HPV 18-positive cases. These results were consistent with previous studies, showing that even if the cytology was NILM, HPV 16/18-positive women still conferred a relatively high risk of CIN 3+. [13, 24] Therefore, this study supported the strategy of HPV-16/18 genotyping for women with HPV-positive and cytology-negative cytology in the Chinese population.

## Limitations

The study had some limitations. First, the research was conducted in a single institution retrospectively. Hence the conclusions may not be generalized to the whole Chinese population without caution. Second, the number of cases was relatively low for coinfection of HPV 16/18 and several HPV genotypes. Third, medical records were incomplete for some patients, and there were no records of past history, smoking history, age at first intercourse, number of sexual partners, especially HPV vaccination status. However, a nationwide survey demonstrated that the HPV vaccine uptake rate of females was only 3% in mainland China [37], hence it is reasonable to infer that most of the included women were not vaccinated against HPV. Fourth, two types of HPV genotyping tests may result in bias, but they have been well verified and reached a high agreement [19–23].

## Conclusion

In this cross-sectional study of HPV 16/18-positive women, the effects of multiple infection were likely complicated and varied with specific HPV genotypes. Generally, HPV16 co-infected with other genotypes of HPV except HPV18 was associated with decreased risk of CIN 3 + independently. More attention should be paid to the effects of multiple infection. Besides, Cytologic results were informative when HPV 16/18 was positive. Cytologic abnormalities in HPV 16/18 infected women were associated with a greater risk of CIN 3+, especially ASC-H and HSIL+. In the case of HPV 16/18 infection and HSIL + in the Chinese population, expedited treatment might be acceptable.

### Supplementary Information

**Additional file1**. **Table S1**: Patients characteristics. **Table S2**: Binary logistic regression analysis of HPV 16/18 infection patterns, adjusted by age, vaginal bleeding, and cytology results (referring to Single HPV 18 infection). **Figure S1**: Prevalence of CIN 3+ in HPV 16/18-positive women stratified by age (n = 7940).

## References

[1] de Sanjose S, Quint W, Alemany L (2010). Human papillomavirus genotype attribution in invasive cervical cancer: a retrospective cross-sectional worldwide study. Lancet Oncol.

[2] Perkins R, Guido R, Castle P (2020). 2019 ASCCP risk-based management consensus guidelines for abnormal cervical cancer screening tests and cancer precursors. J Low Genit Tract Dis.

[3] Wright T, Stoler M, Behrens C, Apple R, Derion T, Wright T (2012). The ATHENA human papillomavirus study: design, methods, and baseline results. Am J Obstet Gynecol.

[4] Zhang J, Zhao Y, Dai Y (2021). Effectiveness of high-risk human papillomavirus testing for cervical cancer screening in china: a multicenter, open-label. Random Clin Trial JAMA Oncol.

[5] Fontham ETH, Wolf AMD, Church TR (2020). Cervical cancer screening for individuals at average risk: 2020 guideline update from the American Cancer Society. CA Cancer J Clin.

[6] Wu P, Xiong H, Yang M (2019). Co-infections of HPV16/18 with other high-risk HPV types and the risk of cervical carcinogenesis: a large population-based study. Gynecol Oncol.

[7] Bao H, Jin C, Wang S (2021). Prevalence of cervicovaginal human papillomavirus infection and genotypes in the pre-vaccine era in China: a nationwide population-based study. J Infect.

[8] Sjoeborg KD, Tropé A, Lie AK (2010). HPV genotype distribution according to severity of cervical neoplasia. Gynecol Oncol.

[9] Bello B, Spinillo A, Alberizzi P (2009). Cervical infections by multiple human papillomavirus (HPV) genotypes: prevalence and impact on the risk of precancerous epithelial lesions. J Med Virol.

[10] Dickson EL, Vogel RI, Geller MA, Downs LS (2014). Cervical cytology and multiple type HPV infection: a study of 8182 women ages 31–65. Gynecol Oncol.

[11] Carrillo-García A, Ponce-de-León-Rosales S, Cantú-de-León D (2014). Impact of human papillomavirus coinfections on the risk of high-grade squamous intraepithelial lesion and cervical cancer. Gynecol Oncol.

[12] Chaturvedi AK, Katki HA, Hildesheim A (2011). Human papillomavirus infection with multiple types: pattern of coinfection and risk of cervical disease. J Infect Dis.

[13] Demarco M, Egemen D, Raine-Bennett TR (2020). A study of partial human papillomavirus genotyping in support of the 2019 ASCCP risk-based management consensus guidelines. J Low Genit Tract Dis.

[14] Stoler M, Wright T, Parvu V (2019). HPV testing with 16, 18, and 45 genotyping stratifies cancer risk for women with normal cytology. Am J Clin Pathol.

[15] Monsonego J, Cox JT, Behrens C (2015). Prevalence of high-risk human papilloma virus genotypes and associated risk of cervical precancerous lesions in a large U.S. screening population: data from the ATHENA trial. Gynecol Oncol.

[16] Xu H, Liu Y, Luo Y (2021). The risk stratification for cervical cancer and precursors of domestic HPV testing with HPV 16/18 genotyping in women with NILM cytology in CentralChina: a cohort study. Front Oncol.

[17] Tao X, Zhang H, Li J (2019). Prevalence of HPV-16/18 genotypes and immediate histopathologic correlation results in a Chinese population with negative cytology and positive high-risk HPV testing. Cancer Cytopathol.

[18] Chan P, Cheung T, Li W (2012). Attribution of human papillomavirus types to cervical intraepithelial neoplasia and invasive cancers in Southern China. Int J Cancer.

[19] Zeng Z, Yang H, Li Z (2016). Prevalence and Genotype Distribution of HPV Infection in China: analysis of 51,345 HPV genotyping results from China's Largest CAP certified laboratory. J Cancer.

[20] Jiang W, Austin RM, Zhang H (2022). The clinical utility of extended high-risk HPV genotyping in women with ASC-US cytology. Am J Clin Pathol.

[21] Sun P, Song Y, Ruan G (2017). Clinical validation of the PCR-reverse dot blot human papillomavirus genotyping test in cervical lesions from Chinese women in the Fujian province: a hospital-based population study. J Gynecol Oncol.

[22] Tang X, Jones TE, Jiang W (2023). Extended human papillomavirus genotype distribution in cervical intraepithelial neoplasia and cancer: analysis of 40 352 cases from a large academic gynecologic center in China. J Med Virol.

[23] Liao G, Jiang X, She B (2020). Multi-infection patterns and co-infection preference of 27 human papillomavirus types among 137,943 gynecological outpatients across China. Front Oncol.

[24] Tao X, Zhang H, Wang S (2021). Prevalence and carcinogenic risk of high-risk human papillomavirus subtypes in different cervical cytology: a study of 124,251 cases from the largest academic center in China. J Am Soc Cytopathol.

[25] Quint W, Jenkins D, Molijn A (2012). One virus, one lesion–individual components of CIN lesions contain a specific HPV type. J Pathol.

[26] van der Marel J, Quint W, Schiffman M (2012). Molecular mapping of high-grade cervical intraepithelial neoplasia shows etiological dominance of HPV16. Int J Cancer.

[27] Salazar KL, Zhou HS, Xu J (2015). Multiple human papilloma virus infections and their impact on the development of high-risk cervical lesions. Acta Cytol.

[28] Schmitt M, Depuydt C, Benoy I (2013). Multiple human papillomavirus infections with high viral loads are associated with cervical lesions but do not differentiate grades of cervical abnormalities. J Clin Microbiol.

[29] Cuschieri KS, Cubie HA, Whitley MW (2004). Multiple high risk HPV infections are common in cervical neoplasia and young women in a cervical screening population. J Clin Pathol.

[30] Wentzensen N, Nason M, Schiffman M (2014). No evidence for synergy between human papillomavirus genotypes for the risk of high-grade squamous intraepithelial lesions in a large population-based study. J Infect Dis.

[31] Wheeler CM, Hunt WC, Schiffman M, Castle PE (2006). Human papillomavirus genotypes and the cumulative 2-year risk of cervical precancer. J Infect Dis.

[32] Sundstrom K, Ploner A, Arnheim-Dahlstrom L (2015). Interactions between high- and low-risk HPV types reduce the risk of squamous cervical cancer. J Natl Cancer Inst.

[33] Pinto LA, Viscidi R, Harro CD (2006). Cellular immune responses to HPV-18, -31, and -53 in healthy volunteers immunized with recombinant HPV-16 L1 virus-like particles. Virology.

[34] Lin W, Chen B, Wu B (2021). Cervical cancer screening rate and willingness among female migrants in Shenzhen, China: three-year changes in citywide surveys. Cancer Res Treat.

[35] Ma Y, Di J, Bi H (2020). Comparison of the detection rate of cervical lesion with TruScreen, LBC test and HPV test: a real-world study based on population screening of cervical cancer in rural areas of China. PLoS ONE.

[36] Bao H, Zhang L, Wang L (2018). Significant variations in the cervical cancer screening rate in China by individual-level and geographical measures of socioeconomic status: a multilevel model analysis of a nationally representative survey dataset. Cancer Med.

[37] Hu S, Xu X, Zhang Y (2021). A nationwide post-marketing survey of knowledge, attitude and practice toward human papillomavirus vaccine in general population: implications for vaccine roll-out in mainland China. Vaccine.

