# Supplementary material for: Risk of cervical intraepithelial neoplasia grade 3 or more diagnoses for human papillomavirus16/18-positive women by cytology and co-infection status
Source: Infect Agent Cancer. 2023 Oct 9;18:57. doi: 10.1186/s13027-023-00540-9 (PMC10561498; doi:10.1186/s13027-023-00540-9)
Supplement: Supplementary file 1 — Additional file1. Table S1: Patients characteristics. Table S2: Binary logistic regression analysis of HPV 16/18 infection patterns, adjusted by age, vaginal bleeding, and cytology results (referring to Single HPV 18 infection). Figure S1: Prevalence of CIN 3+ in HPV 16/18-positive women stratified by age (n = 7940). [file 13027_2023_540_MOESM1_ESM.docx]

**Table S1** Patients characteristics

**Table S2** Binary logistic regression analysis of HPV 16/18 infection patterns, adjusted by age, vaginal bleeding, and cytology results (referring to Single HPV 18 infection).

**Figure S1** Prevalence of CIN 3+ in HPV 16/18-positive women stratified by age (n = 7940).

**Table S1 Patients characteristics**

| **characteristics** | **Total, No. (%)** |
| --- | --- |
| **Age, median (range)** | 40 (25-84) |
| **Vaginal bleeding** |  |
| **No** | 7352 (92.6%) |
| **Yes** | 588 (7.4%) |
| **HPV 16 (+)** | 6211 (78.2%) |
| **Multiple HPV infection** | 2051 (2051/6211, 33.0%) |
| **Single HPV infection** | 4160 (4160/6211, 67.0%) |
| **HPV 18 (+)** | 1895 (23.9%) |
| **Multiple HPV infection** | 827 (827/1895, 43.6%) |
| **Single HPV infection** | 1068 (1068/1895, 56.4%) |
| **Subjects with Cytology results** | 6533 (82.3%) |
| **Grade** |  |
| **Normal** | 4028 (50.7%) |
| **CIN 1** | 956 (12.0%) |
| **CIN 2** | 840 (10.6%) |
| **CIN3/AIS** | 1430 (18.0%) |
| **Cancer** | 686 (8.6%) |
| **Total** | 7940 (100%) |

HPV, human papillomavirus; CIN, cervical intraepithelial neoplasia; AIS, adenocarcinoma in situ.

**Table S2 Binary logistic regression analysis of HPV 16/18 infection patterns, adjusted by age, vaginal bleeding, and cytology results (referring to Single HPV 18 infection).**

| **HPV infection patterns** | **CIN 3+ vs.** $\text{≤}$**CIN 2** |
| --- | --- |
|  | **OR (95% CI)** |
| **Single HPV 18^a^** | reference |
| **HPV 18 + other hrHPVs** | 0.699 (0.443-1.102) |
| **HPV 18 + lrHPVs** | 0.962 (0.442-2.096) |
| **HPV 18 + lrHPVs + other hrHPVs** | 0.641 (0.280-1.468) |
| **HPV 16 only** | **3.056 (2.381-3.992)** |
| **HPV 16 + other hrHPVs** | **1.899 (1.422-2.536)** |
| **HPV 16 + lrHPVs** | **1.896 (1.253-2.869)** |
| **HPV 16 + lrHPVs + other hrHPVs** | 0.757 (0.457-1.253) |
| **HPV 18 + HPV 16** | **2.284 (1.079-4.883)** |
| **HPV 18 + HPV 16 + other hrHPVs** | 2.168 (0.958-4.907) |
| **HPV 18 + HPV 16 + lrHPVs** | NA |
| **HPV 18 + HPV 16 + lrHPVs + other hrHPVs** | 1.131 (0.229-5.586) |

^a^ For HPV infection patterns, this analysis took single HPV 18 infection as a reference.

HPV, human papillomavirus; HPV, human papillomavirus; other hrHPVs, high-risk HPV 26/31/33/35/39/45/51/52/53/56/58/59/66/68/73/82; lrHPVs, low-risk HPV 6/11/40/42/43/44/55/61/81/83; CIN 3+, cervical intraepithelial neoplasia grade 3 or more severe diagnoses; OR, odds ratio; CI, confidence interval. NA, not applicable.

**Figure S1 Prevalence of CIN 3+ in HPV 16/18-positive women stratified by age (n = 7940).**


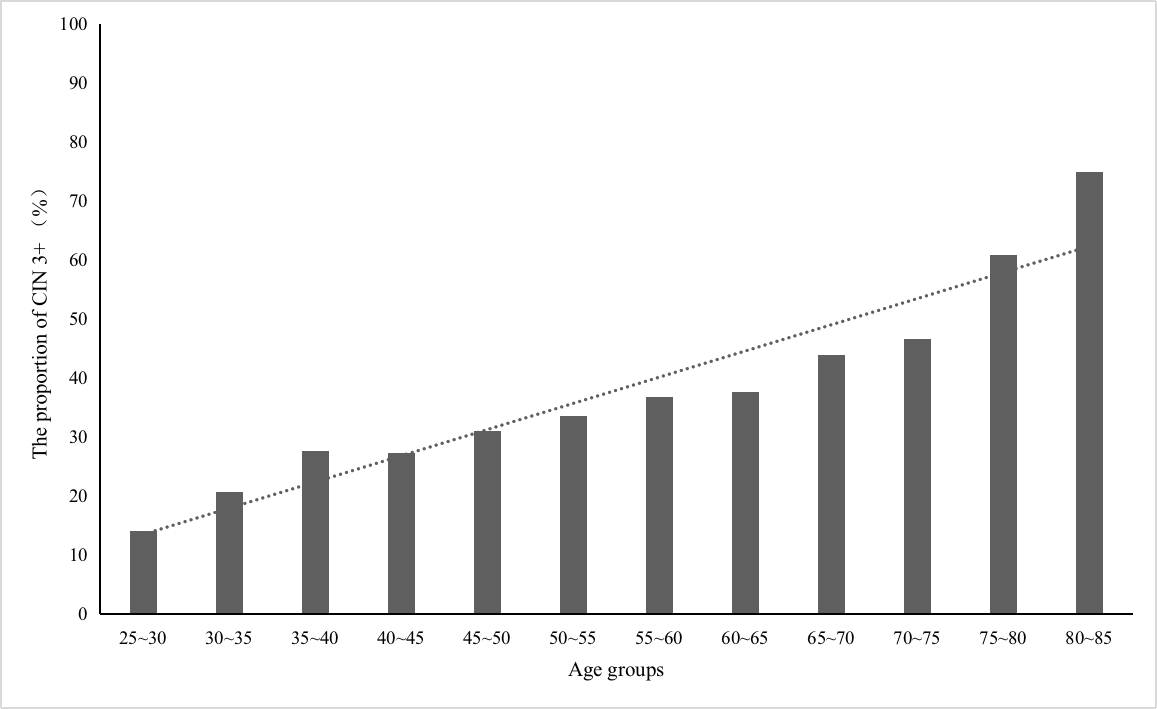


HPV, human papillomavirus; CIN 3+, cervical intraepithelial neoplasia 3 or worse.
